# An Overview on Identification and Regulatory Mechanisms of Long Non-coding RNAs in Fungi

**DOI:** 10.3389/fmicb.2021.638617

**Published:** 2021-04-28

**Authors:** Juan Li, Xiaoying Liu, Ziyu Yin, Zhihong Hu, Ke-Qin Zhang

**Affiliations:** State Key Laboratory for Conservation and Utilization of Bio-Resources in Yunnan, Yunnan University, Kunming, China

**Keywords:** lncRNAs, eukaryotic fungi, transcriptional interference, chromatin remodeling, RNA surveillance

## Abstract

For decades, more and more long non-coding RNAs (lncRNAs) have been confirmed to play important functions in key biological processes of different organisms. At present, most identified lncRNAs and those with known functional roles are from mammalian systems. However, lncRNAs have also been found in primitive eukaryotic fungi, and they have different functions in fungal development, metabolism, and pathogenicity. In this review, we highlight some recent researches on lncRNAs in the primitive eukaryotic fungi, particularly focusing on the identification of lncRNAs and their regulatory roles in diverse biological processes.

## Introduction

Recent researches on non-coding RNAs (ncRNAs) have expanded our knowledge of gene transcriptional regulation. These ncRNAs have been classified mainly into small ncRNAs and long non-coding RNAs (lncRNAs). Small ncRNAs are range from 17 nt to approximately 200 nt in length, while lncRNAs have lengths greater than 200 nt (Wilusz et al., 2009; Pasquinelli, 2012; Rinn and Chang, 2012; Fatica and Bozzoni, 2014). Small RNAs, including microRNAs, small interfering RNAs (siRNAs), and PIWI-interacting RNAs (piRNAs), usually function as negative regulators that interfere with the expression of target RNAs, which to regulate diverse cellular, developmental, and physiological processes (He and Hannon, 2004; Bushati and Cohen, 2007; Carthew and Sontheimer, 2009; Ghildiyal and Zamore, 2009; Czech and Hannon, 2011). In comparison with small ncRNAs, lncRNAs have more complicated and flexible regulatory functions in numerous biological processes, including dosage compensation, alternative splicing, genomic imprinting, X-chromosome inactivation, and so on (reviewed in Wilusz et al., 2009; Hung and Chang, 2010; Lee, 2012; Cheetham et al., 2013; Kung et al., 2013; Fatica and Bozzoni, 2014). Like mRNAs, many lncRNAs are 5’-capped and 3’-polyadenylated. They are usually spliced products of RNA polymerase II (RNAPII). lncRNAs have been classified as sense, antisense, bidirectional, intronic, and intergenic based on their locations relative to adjacent encoding genes (Mishra and Kanduri, 2019). Regulatory lncRNAs are often expressed at specific development stages or in response to conditional changes of nutrition or environment (reviewed in Hung and Chang, 2010; Guttman and Rinn, 2012; Lee, 2012; Rinn and Chang, 2012). Also, some lncRNAs are translated into stable functional micropeptides, or function as a sponge to recruit microRNAs (Anderson et al., 2015; Paraskevopoulou and Hatzigeorgiou, 2016; Yeasmin et al., 2018; Zhao et al., 2020). Some lncRNAs form circlular RNAs (circRNAs) and act as transcript effectors that regulate—the target gene expression (Cesana et al., 2011; Sanchez-Mejias and Tay, 2015; Thomson and Dinger, 2016; Zhong et al., 2018). All these researches have enhanced our understanding about the functions of non-coding RNAs in different kinds of biological processes.

At present, a lot of lncRNAs such as *H19, Xist*, *MALAT1*, and *HOTAIR* have been well characterized in mammals through genetic and molecular studies (Brown et al., 1991; Tripathi et al., 2010; Hung et al., 2011; Nagano and Fraser, 2011). In plants, since the first plant lncRNA *Enod40* was discovered in 1994 (Crespi et al., 1994), several plant lncRNAs have also been functional identified (Franco-Zorrilla et al., 2007; Swiezewski et al., 2009; Ariel et al., 2014, 2020; Kim and Sung, 2017; Wu et al., 2020). These lncRNAs from mammals and plants can regulate gene expression at epigenetic, transcription, and post-transcription levels and widely take part in various physiological and pathological processes (reviewed in Rinn and Chang, 2012; Cheetham et al., 2013; Fatica and Bozzoni, 2014). However, research on the mechanisms of lncRNAs functions in eukaryotic microbes is still in its infancy. Although 80% of budding and fission yeast genomes are transcribed and most of the transcripts are translated into proteins, many non-coding transcripts still exist (Christie et al., 2004; David et al., 2006; Nagalakshmi et al., 2008; Jacquier, 2009). With the development of advanced biotechnologies, such as high-resolution tilling arrays and high-throughout sequencing (e.g., RNA-Seq), numerous lncRNAs have also been found in several fungi. Although the molecular functions of most fungal ncRNAs remaining elusive, a small number of functions in model organisms, such as *Saccharomyces cerevisiae*, *Schizosaccharomyces pombe*, and *Neurospora crassa*, have been determined. These fungal lncRNAs have different functions in a wide variety of biological processes, including fungal development, mating-type regulation, metabolism, cell differentiation, sporulation, and nutrient metabolism (Donaldson and Saville, 2012). Because of the vital roles of fungi in ecosystems, studying their lncRNAs may help us expand our knowledge of ncRNA-related mechanisms in different species. In this review, we provide a recent snapshot of lncRNAs in eukaryotic microbes. We aim to describe the regulatory functions of fungal lncRNAs in gene regulation and development.

## Identification and Classification of lncRNAs in Fungi

Advancements in biotechnologies have led to the identification of non-coding transcripts in fungi. Initially, in 2006, tiling arrays were used to identify the lncRNAs of the *S. cerevisiae* genome (David et al., 2006; Samanta et al., 2006). However, this method produced a high level of background noise and was difficult to interpret with a high degree of confidence. Thus, with the advancement of next-generation sequencing, strand-specific RNA sequencing has been successfully used to identify lncRNAs in different fungal organisms, including *S. cerevisiae*, *S. pombe*, and *N. crassa.* At present, the RNA-seq technique is the most powerful method to identify fungal lncRNAs because it reveals both already known sequences and novel variants (Piskol et al., 2013). Furthermore, lncRNA microarrays, which is different from conventional mRNA sequence-based gene expression microarrays, have been developed to assess the biological relevance of lncRNAs in pathological conditions (Huang et al., 2018). Using high-throughput sequencing and bioinformatics methods, putative ncRNAs are obtained. Moreover, several biological molecular methods have provided powerful platforms for detecting the transcriptional regulatory functions of lncRNAs (McDonel and Guttman, 2019). For example, chromatin immunoprecipitation (ChIP) and related derivative technologies, such as RIP (RNA immunoprecipitation), CLIP (UV crosslinking and immunoprecipitation), ChIRP (chromatin isolation by RNA purification), CHART (capture hybridization analysis of RNA targets), and RAP (RNA antisense purification), have been used to investigate the actions of lncRNAs that interact with RNA, DNA, proteins, and nucleic acids (Cao et al., 2019). Additionally, techniques such as genetic modification, overexpression or knockdown strategies, and phenotype analyses have been used to determine the functional importance of lncRNAs in different species.

In general, lncRNAs are present at relatively low abundance levels and are not effectively detected using empirical techniques because they likely exist in a dynamic equilibrium that is constantly monitored by the RNA surveillance machinery (Nair et al., 2020). The RNA surveillance system monitors each step of RNA biogenesis, including 5′-capping, 3′-splicing end formation, polyadenylation, nuclear export, and full-length protein translation (Pefanis et al., 2015; Nair et al., 2020). In yeast, RNA surveillance-mediated RNA degradation is mainly classified as nuclear and cytoplasmic degradation. In the nucleus, the RNA exosome complex has critical functions in the 3′–5′ RNA degradation associated with early transcription termination (Houseley et al., 2006; Schmid and Jensen, 2008; Fraga de Andrade et al., 2020). The eukaryotic exosome complex includes nine subunits: six distinct proteins form as a “ring” and three RNA-binding proteins form a “cap.” Interestingly, two additional subunits, Dis3 (Rrp44) and Rrp6 (Exosc10), provide the enzymatic activity of the exosome complex (Houseley et al., 2006; Januszyk and Lima, 2010; Wasmuth and Lima, 2012). Rrp6 is a nuclear-specific 3′–5′ distributive exoribonuclease (Lykke-Andersen et al., 2009; Wasmuth et al., 2014). In addition, efficient RNA degradation by the exosome requires the polyadenylation of the Trf4-Air2-Mtr4p (TRAMP) complex (Houseley et al., 2006). Although the eukaryotic RNA exosome complex functions in both the nucleus and the cytoplasm, studies have found that the transcripts in the latter were mainly degraded by the 5′–3′ exonuclease Xrn1 (Nagarajan et al., 2013). Before the Xrn1-mediated degradation, Dcp2 needs to remove the 5′-caps (Garneau et al., 2007).

Eliminating key components of the RNA surveillance system has enabled researchers to identify some previously undescribed lncRNAs in cells. Thus, a series of novel lncRNAs has been identified in fungi. For example, 925 transcripts encoded in intergenic regions accumulate in *S. cerevisiae* mutants lacking the exosome subunit Rrp6, while limited expression changes have been identified in most of the open reading frame transcripts in both wild-type and Rrp6 mutant (Wyers et al., 2005). These identified non-coding transcripts are transcribed by RNAPII, capped, and polyadenylated. More importantly, they lack promoter elements and the rapid degradation by nuclear exosomes found in wild-type cells. Consequently, these ncRNAs are referred to as cryptic unstable transcripts (CUTs) (Table 1; Wyers et al., 2005; Berretta et al., 2008). The high-resolution genomic map of yeast revealed 1,496 CUTs, and numerous new CUTs may be associated with regulatory mechanisms (Neil et al., 2009). Another full inactivation of the exosome resulted in at least 1,600 CUTs (Gudipati et al., 2012). Interestingly, aberrant polyadenylated transcripts from small nucleolar RNA genes also accumulate in Rrp6 mutants (Davis and Ares, 2006). After deleting the decapping enzyme Dcp2, more than 100 lncRNAs accumulate in *S. cerevisiae*, indicating that decapping also control the stability of lncRNAs (Ramaiah et al., 2012). Also, a kind of cryptic non-coding transcripts named *Xrn1*-sensitive unstable transcripts (XUTs) have been observed with the absence of *Xrn1* in budding yeast cells (Table 1). XUTs can modulate the chromatin structure of the promoter regions to repress the expression of sense transcripts (van Dijk et al., 2011). It is interesting that both the Dcp2-sensitive lncRNAs and XUTs are often antisense to encoding genes.

**TABLE 1:** Classification of lncRNAs in fungi.

Name	Related enzyme or protein	How to found	Functions	References
CUTs	Rrp6 (3′–5′ distributive exoribonuclease)	With the inactivation of Rrp6	Nuclear, regulatory mechanisms	Wyers et al., 2005; Gudipati et al., 2012
Dcp2-sensitive lncRNAs	Dcp2 (decapping enzyme of 5′-caps)	With the inactivation of Dcp2	Cytoplasm, function unknown	Ramaiah et al., 2012
XUTs	Xrn1 (5′–3′exonuclease)	With the inactivation of Xrn1	Cytoplasm, modulate the chromatin structure of the promoter regions to repress the expression of sense transcript	van Dijk et al., 2011
SUTs	May be substrates of Xrn1	Expressed in vegetative yeast cells	Regulate vegetative growth	Xu et al., 2009
MUTs	Most degraded by the exosome component Rrp6	Expressed during Mitotic growth	Regulate the sense genes during meiosis	Lardenois et al., 2011
TERRAs	Rat1(5′-end exonuclease)	Degraded by Rat1	Control telomere length	Luke et al., 2008
CD-CUTs	Cytoplasmic non-sense-mediated mRNA decay pathway	With the inactivation of cytoplasmic non-sense-mediated mRNA decay pathway	Interferes with the binding of RNAPII and transcriptional activators by repressing bona fide promoters under repressive conditions.	Toesca et al., 2011
NUTs	Nrd1 (RNA-binding factor)	With the mutant of Nrd1	Sensitive to the early Termination of lncRNA transcription	Schulz et al., 2013
Set2-repressed antisense transcripts	Set2 (histone methyltransferase)	With the deletion of Set2	Undetermined	Venkatesh et al., 2016

Beside the identification of those non-coding RNAs degraded by the RNA surveillance system, a new set of transcripts, named stable unannotated transcripts (SUTs), which are expressed with low abundance in vegetative yeast cells, have also been identified (Table 1; Xu et al., 2009). Interestingly, most SUTs may be substrates of *Xrn1* because they accumulated in an *Xrn1* mutant grown in a YPD (Yeast Extract Peptone Dextrose) medium (Xu et al., 2009). Furthermore, non-coding transcripts have not only been revealed during vegetative growth but also during mitotic growth. Meiotic unannotated transcripts (MUTs), which accumulate during mitotic growth in *S. cerevisiae*, have also been discovered recently (Table 1; Lardenois et al., 2011). Some MUTs are transcribed on the antisense strands of coding genes, implying that they may regulate the sense genes during meiosis. Additionally, most MUTs are degraded by the exosome component Rrp6, suggesting that they are a subclass of CUTs (Lardenois et al., 2011).

Interestingly, a pervasive yeast telomeric repeat-associated cryptic ncRNAs (*TERRAs*), which is degraded by the nuclear 5′-end exonuclease Rat1, has also been found to control telomere length (Table 1; Luke et al., 2008). Recently, a group of cryptic transcripts, named cytoplasmically degraded-CUTs (CD-CUTs), have been found in with the inactivation of the cytoplasmic non-sense-mediated mRNA decay pathway (Table 1; Toesca et al., 2011). The transcription of these CD-CUTs interferes with the binding of RNAPII and transcriptional activators by repressing bona fide promoters under repressive conditions.

Thus, identifying ncRNAs involves surveying stabilized or enriched RNAs in mutants defective in RNA degradation pathway provides novel information on the genome-wide occupancy of the transcriptional machinery. In addition, a class of ncRNAs termed Nrd1-unterminated transcripts (NUTs), which is sensitive to the early termination of lncRNA transcription, has also been identified in the RNA-binding factor Nrd1 mutant cells (Table 1; Schulz et al., 2013). Similarly, Set2-repressed antisense transcripts have been identified using the deletion of the histone methyltransferase Set2 (Table 1; Venkatesh et al., 2016). However, although it still not clear whether these unstable transcripts form explicit mechanisms or represent an unexpected side effect of transcriptome surveillance, these types of unstable transcripts result in the silencing of their target genes. Thus, they are usually used to control pervasive transcription and offer protection from gene silencing throughout the life cycle.

## Regulatory Mechanisms of lncRNAs in Fungi

In general, functional lncRNAs can bind with DNA, RNA, or protein to regulate the expression of target genes through transcriptional interference, promoter occlusion, and/or recruit epigenetic chromatin-modifier recruitment via *cis*- or *trans*- model (Mishra and Kanduri, 2019). Because *S. cerevisiae* lacks apparent homologs of Argonaut or Piwi-like proteins, those complexes, such as RNA-induced silencing complex (RISC) and RNA-induced transcriptional silencing (RITS) which mediate the interactions between regulatory siRNAs and their nucleotide targets, have not been observed in this species (Aravind et al., 2000; Drinnenberg et al., 2009, 2011). However, *S. cerevisiae* may have evolved novel post-transcriptional regulatory strategies to adapt to the loss of RNAi. For example, it can utilize the sequence-specific *Ty1* retrotransposon to regulate the ncRNA-mRNA/DNA interactions (Berretta et al., 2008). In *S. pombe*, siRNAs are incorporated into RNA-induced transcriptional silencing complexes. This complex then recruit the histone methyltransferase Clr4 and the *S. pombe* homolog of metazoan heterochromatin protein Swi6 to promote the transcriptionally silent heterochromatin formation (Moazed et al., 2006; Zhang et al., 2011). Moreover, a novel RNAi-independent cosuppression of long terminal repeat-retroelements have also been found in the phytopathogenic fungus *Magnaporthe oryzae* (Murata et al., 2007). In addition, lncRNAs such as *meiRNAs*, *TERRAs*, and telomerase RNAs can act as scaffolds or attract proteins to alter their functions in yeast (Ding et al., 2012; Cusanelli and Chartrand, 2015). They are presented in detail in the following sections.

Depending on their modes of gene regulation, lncRNAs may also be categorized as *cis-* or *trans-*acting (Camblong et al., 2007, 2009). The *trans*-acting lncRNAs often regulate target genes through pre-translational and/or post-translational mechanisms and act as guides or scaffolds for chromatin-remodeling complexes (Guil and Esteller, 2012). However, most of the functional lncRNAs identified in fungi mainly perform their functions in *cis* using different regulatory strategies. At present, the simplest and probably most common regulatory strategy that has been reported among the fungal lncRNAs is transcriptional interference, especially interfering with the transcription of proximally located genes. lncRNAs govern the expression of their adjacent genes, both sense and antisense, in a repressive or activating manner. Also, their regulatory mechanisms are varied, including blocking the transcription machinery, modulating the nucleosomal arrangement, and stimulating regulatory factors binding or dissociation. There are numerous examples of lncRNAs in fungi exerting transcriptional interference, such as *nc-tgp1*, *prt*, and *prt2* (reviewed in Kornienko et al., 2013; Vance and Ponting, 2014; Shuman, 2020). Moreover, numerous functionally identified lncRNAs interact physically with RNAPII complexes to regulate mRNAs transcription (Goodrich and Kugel, 2006).

Another regulatory strategy of lncRNAs is chromatin remodeling. They have been identified as the regulators of chromatin structure because their transcription recruits various histone/DNA modification enzymes, such as methylation of histone 3 (H3K4me3, H3K9me3, H3K27me3, and H3K36me3), to alter chromatin structure and modify histones, which influences the recruitment or activity of transcription factors (Martens et al., 2004, 2005; Hongay et al., 2006; Uhler et al., 2007; Houseley et al., 2008). Chromatin remodeling guided by lncRNAs contributes mechanistically to the establishment of chromatin structure and the maintenance of epigenetic memory. Antisense transcript-mediated chromatin remodeling may occur in *cis* or *trans*. For instance, the antisense transcript *Ty1AS* inhibits its retro-transposition in *S. cerevisiae* in *trans* (Berretta et al., 2008). All the metabolic stress-inducing lncRNA *mlonRNA* from *S. pombe* and *ncASP3* and the antisense lncRNA of the *CDC28* gene in *S. cerevisiae* are involved in stress responses through affecting the chromatin organization (Nadal-Ribelles et al., 2014).

## Functional Diversity of lncRNAs in Fungi

Although numerous long non-coding transcripts have been identified in fungi, only a few have been functionally identified (Table 2). However, these functional studies showed that lncRNAs in fungi are involved in complex regulatory networks, performing essential cellular tasks, including the regulation of meiosis and mating, metabolisms, cell aging, circadian rhythms, and pathogenesis (Table 2). Here, we describe some functionally identified lncRNAs in fungi to provide an understanding of their specific regulatory roles.

**TABLE 2:** Experimentally characterised lncRNAs in fungi.

Species	Non-coding	Gene	Antisense/sense	Cis/trans	Coactivator	Cellular	Regulatory	References
	**RNA name**	**name**	**to mRNA**	**regulation**		**process**	** mechanism**	
*S. cerevisiae*	*GAL10-ncRNA*	*GAL10/GAL1*	Antisense/Sense	*cis*	reb1	Galactose utilization	Histone modification	Houseley et al., 2008
*S. cerevisiae*	*GAL4 lncRNA*	*GAL4*	Antisensense	*cis*	/*	Galactose utilization	/	Geisler et al., 2012
*S. cerevisiae*	*ncASP3*	*ASP3*	Sense	*cis*	/	Nitrogen starvation	Histone modification, chromatin remodeling	Huang et al., 2010
*S. cerevisiae*	*PHO84 antisense transcripts*	*PHO84*	Antisense	*trans, cis*	Hda1/2/3	Phosphate metabolism, Cell aging	Histone modification	Camblong et al., 2007
*S. cerevisiae*	*REM2*	*IME4*	Antisense	*cis*	/	Sexual differentiation and mating-type control	Transcriptional interference	Hongay et al., 2006
*S. cerevisiae*	*IRT1*	*IME1*	Sense	*cis*	Rem1	Sexual differentiation and mating-type control	Histone modification, transcriptional interference	van Werven et al., 2012
*S. cerevisiae*	*REM3*	*ZIP2*	Antisense	*cis*	/	Sexual differentiation and mating-type control	Transcription interference	Gelfand et al., 2011
*S. cerevisiae*	*SRG1*	*SER3*	Sense	*cis*	SAGA and Swi/Snf, Cha4,	Serine synthesis	Transcriptional interference	Martens et al., 2005
*S. cerevisiae*	*pHO-lncRNA*	*HO*	Sense	*cis*	/	Mating type interconversion, cell-cycle	Transcriptional interference, nocleosome repositioning	Yu et al., 2016
*S. cerevisiae*	*PWR1*	*FLO11*	Antisense	*cis*	/	Cell-cell adhesion	Transcriptional interference	Bumgarner et al., 2009
*S. cerevisiae*	*ICR1*	*FLO11*	Sense	*cis*	/	Cell-cell adhesion	Promoter occlusion, silencing	Bumgarner et al., 2009
*S. cerevisiae*	*AS-PHO5*	*PHO5*	Antisense	*cis*	/	Phosphate metabolism	Activate PHO5 transcription during phosphate starvation	Uhler et al., 2007
*S. cerevisiae*	*Antisense lncRNA of CDC28*	*CDC28*	Antisense	*cis*	/	Osmostress	Chromatin remodeling	Nadal-Ribelles et al., 2014
*S. cerevisiae*	*Antisense lncRNA of Ty1*	*Ty1*	Antisense	*trans*	/	Transponson silencing	RNA-interference	Berretta et al., 2008
*S. cerevisiae*	*TERRA*	*Telomerase RNA*	/	*/*	/	Telomere replication	Regulation of telomerase activity, heterochromatin formation	Luke et al., 2008
*S. cerevisiae*	*TLC1*	*Telomerase RNA*	/	*/*	Est1, Est2, and Est3	Telomere replication	Scaffold for telomerase complex	Gallardo et al., 2008
*S. cerevisiae*	*ADF1*	*MDF1*	Antisense	*cis*	/	Vegetative growth	Transcriptional suppression	Li et al., 2010
*S. pombe*	*prt/nc-pho1*	*pho1*	Sense	*cis*	Pho7	Phosphate metabolism.	Chromatin remodeling	Chatterjee et al., 2016
*S. pombe*	*nc-tgp1*	*tgp1*	Sense	*cis*	Pho7	Phosphate metabolism	Transcriptional interference	Ard et al., 2014
*S. pombe*	*prt2*	*pho84(prt/pho1)*	Sense	*cis*	/	Phosphate metabolism	Transcriptional interference	Garg et al., 2018
*S. pombe*	*mlonRNAs*	*fbp1*	Sense	*cis*	United States1, United States2	Glucose starvation	Chromatin remodeling	Hirota et al., 2008
*S. pombe*	*SPNCRNA .1164*	*atf1*	/	*trans*	/	Oxidative stress	Activation	Leong et al., 2014
*S. pombe*	*meiRNA-S and L*	*sme2*	Sense	*cis*	Mei2, Mmi1	Meiosis	Decoy of Mmi1	Shichino et al., 2014
*S. pombe*	*TER1*	*telomerase RNA*	/	*/*	/	Telomere replication	Scaffold for telomerase complex	Leonardi et al., 2008
*T.reesei*	*HAX1*	*cellulase genes*	/	*trans*	Xyr1	Cellulose metabolism	Activation	Till et al., 2018
*C. heterost rophus.*	*Antisense of tramscription factor CMR1*	Melanin gene cluster	Antisense	*trans*	MAPK pathway	Melanin biosynthesis	Regulate the transition of the melanin gene cluster	Eliahu et al., 2007
*N. crassa*	*qrf*	*frq*	Antisense	*cis*	/	Rhythmic conidiation	Chromatin modifications and the premature termination of transcription	Kramer et al., 2003
*U. maydis*	*Antisense to gene um02151*	*um02151*	Antisense	/	/	Pathogenesis	Unknown	Donaldson and Saville, 2013
*C. neoformans*	*RZE1*	*Znf2*	/	/	/	Yeast-to-hypha transition	Unknown	Chacko et al., 2015
*F. oxysporum*	*Fo-carP*	*carS*	/	/	/	Carotenoid biosynthesis	Active expression of the carotenoid genes	Parra-Rivero et al., 2020
*F. fujikuroi*	*Ff-carP*		/	/	/			

*Details unknown.

## Cell Cycle or Meiosis Control

**FIGURE 1:**
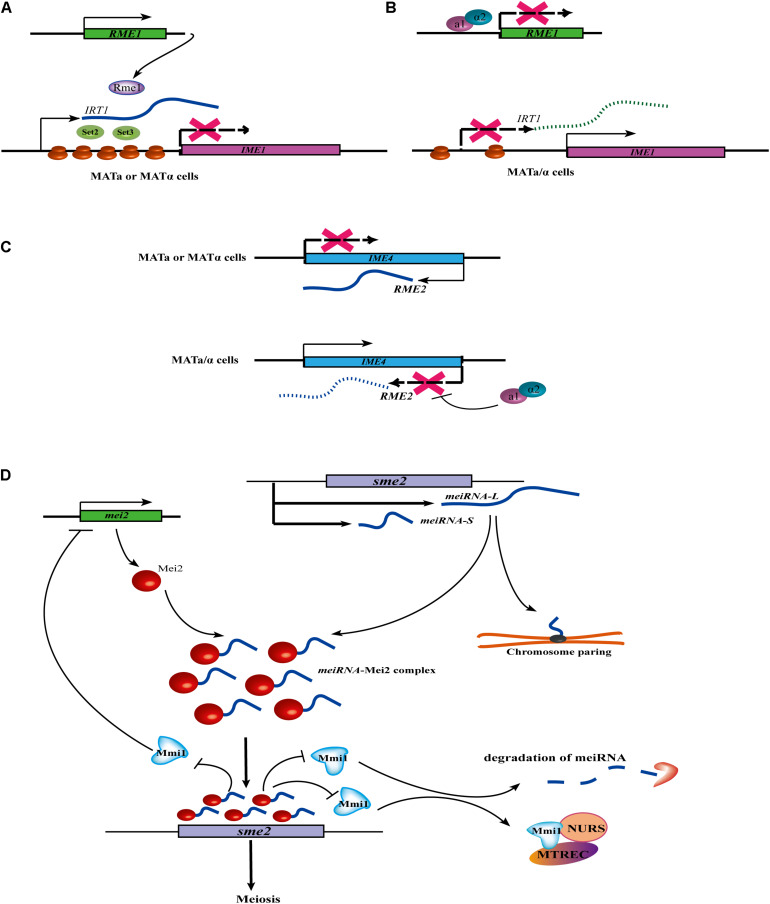
Cell cycle or meiosis control of lncRNA in fungi. **(A)** In *MAT*a or *MAT*α haploid cells, the expression of *IRT1* is induced by a transcription activator Rme1. Then transcribed *IRT1* can recruit the histone methyltransferase Set2 and the histone deacetylase Set3 at the *IME1* promoter, finally leading to the inhibit of *IME1* expression. **(B)** In the homozygous *MAT*a/α diploid cells, the a1/α2 heterodimer can inhibit the expression of *REM1*, so *IRT1* can’t be expressed without the induction of Rme1. *IME1* can successfully transcript without the repression of *IRT1*. **(C)** In MATa or MATα haploid cells, lncRNA *REM2* inhibits transcription elongation of *IME4* to prevent germ cell differentiation. However, in MATa/α diploid cells, the a1/α2 heterodimer represses the transcription of *REM2* by binding at a conserved site located downstream of the *IME4*, thus allowing for induction of *IME4.* **(D)** *meiRNA* regulates the expression of meiotic gene and chromosome pairing. In meiotic cells, two isoforms of the *meiRNA* (i.e., *meiRNA-S* and *meiRNA-L*) are transcribed and form a *meiRNA-*Mei2 complex by interacting with RNA-binding protein Mei2. The Mei2-meiRNA complex blocks the RNA-binding protein Mmi1, thus allowing the *sme2* to be translated, thus initiating meiosis. During meiotic prophase in *S. pombe*, the ends of each chromosome are tied up to the spindle pole body. At the *sme2* loci on chromosome II, the formation of a robustly chromosome pairing is dependent on *meiRNA*. Interestingly, Mmi1 also inhibits Mei2 by forming a double negative feedback loop. In addition, Mmi1 can interact with a nuclear complex called Meiotic gene silencing complex MTREC/NURS MTREC (MTl1-REd1 Core) or NURS (NUclear RNA Silencing) in meiotic transcript elimination.

In yeast, the mating of haploid cells with the opposite mating type (MATa and MATα) can produces MAT a/α diploid cells. In *S. cerevisiae*, the induction of meiosis and sporulation are dependent on the transcriptional activation of the *IME1* (Inducer of Meiosis 1) gene. At present, at least three lncRNAs (*IRT1, RME2, and RME3*) have been identified to control the switch of mating-type in *S. cerevisiae*. *IRT1* arises from the same strand as the *IME1* promoter. A meiosis-repressive transcription activator, *Rme1*, acts as a coactivator to induce the production of the lncRNA *IRT1.* Then, *IRT1* recruits the histone methyltransferase Set2 and the histone deacetylase Set3 to inhibit *IME1* expression through establishing repressive chromatin at the *IME1* promoter (Figure 1A; van Werven et al., 2012). Further evidence has revealed that the a1/α2 heterodimer inhibits the expression of *RME1*. Without the repression of *IRT1*, *IME1* is successfully transcribed (Figure 1B; van Werven et al., 2012). *RME2* is another lncRNA that inhibits the transcriptional elongation of a putative RNA methyltransferase, *IME4*, to prevent germ cell differentiation in MATa or MATα haploid cells. However, in MATa/α diploid cells, the a1/α2 heterodimer binds at a conserved site located downstream of the *IME4*, leading to the repression of *RME2* and the induction of *IME4* (Figure 1C; Hongay et al., 2006). Moreover, *RME3* represses the expression of its adjacent gene, *ZIP2*, which is required for chromosomal pairing during meiosis (Gelfand et al., 2011).

Additionally, the *pHO-lncRNA*, which originates at ∼2,700 bp upstream of the *HO* gene, is responsible for mating-type interconversion during cell-cycle re-entry after a pheromone-dependent arrest in G1. It is induced to force nucleosome repositioning at the locus of the downstream located *HO* endonuclease in *S. cerevisiae* (Yu et al., 2016). The production of a pheromone (i.e., the α-factor) and nucleosome rearrangement is induced with the transcription of *pHO-lncRNA*, leading to the activating signal loss of the Swi4/Swi6 cell-cycle box-binding factor from the *HO* promoter. The displacement of the binding factor prevents the *HO* expression, which blocks mating-type interconversion during re-entry into the cell cycle (Yu et al., 2016).

**FIGURE 2:**
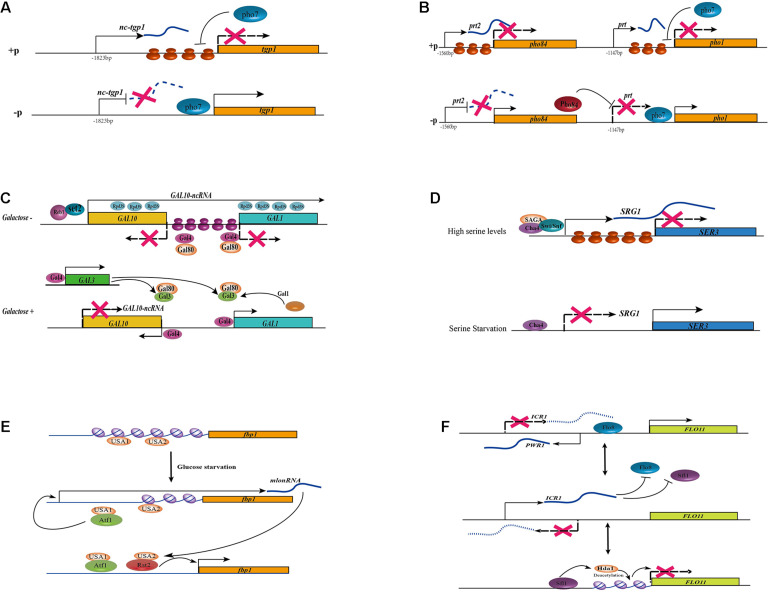
Different regulatory mechanisms of lncRNAs in fungi. **(A)** In the addition of phosphate, the lncRNA *nc-tgp1* initiates at 1,823 nt upstream of the *tgp1* mRNA start site. The nucleosome density at the *tgp1* promoter is increased with the transcription of *nc-tgp1*, which leads to the displacement of Pho7 from the *tgp1* promoters, then results in the derepression of *tgp1.* The transcription of *nc-tgp1* is reduced during phosphate deprivation, leading to a decrease in the nucleosome occupancy and expression of *tgp1.* **(B)** In the addition of phosphate, the lncRNA *prt2* arises from the 1,560 nt upstream of the *pho84* mRNA transcription start site. The transcription of lncRNA *prt2* blocks the expression of its sense gene *pho84*. Similarly, the transcription of lncRNA *prt* blocks the expression of its sense gene *pho1* by increasing nucleosome density at the *pho1* promoter and displacing Pho7 from the *pho1* promoters, leading to the derepression of *pho1*. The expression of lncRNA *prt2* is depressed during phosphate starvation, leading to the production of Pho84, which acts as a repressor of *prt*, finally resulting in the expression of *pho1*. **(C)** lncRNA *GAL10-*ncRNA regulate the expression of *GAL* genes. In the absence of galactose, an antisense transcript *GAL10-*ncRNA that was initiated near the 3’end of *GAL10* and terminated in the *GAL1* coding region. Transcription of this antisense transcript is dependent on the transcriptional activator Reb1 and Set2 methyltransferase and histone deacetylation activities in *cis*, thus resulting in silencing of the whole *GAL* locus. In the absence of galactose, the activity of Gal4 activator is suppressed by Gal80. In the presence of galactose, Gal4 is released, leading to transcriptional activation of the *GAL* gene by sequestering Gal80 with Gal. Both *GAL3* and *GAL1* genes can be activated by Gal4 activator, thus forming two positive feedback loops and leading to stable Gal4 release. **(D)** The transcription of lncRNA *SRG1* represses the adjacent *SER3* gene under serine-rich conditions. When serine is available to the cells, *SRG1* transcription is turned on via a serine-dependent activator Cha4, a coactivator complex SAGA and the ATPase subunit of the Swi/Snf chromatin remodeling complex, leading to the increase in nucleosome occupancy at the SER3 promoter, then repress the expression of *SER*3. In the absence of serine, although the Cha4 constitutively bound to the promoter of lncRNA *SRG1*, its transcription is repressed and the expression of *SER3* is activated. **(E)** lncRNA *mlonRNA* regulates *fbp1* genes through chromatin remodeling. There are two cis-acting regulatory elements in the upstream region of *fbp1*: upstream activating sequence1 (UAS1) and (UAS2). During glucose starvation, UAS1 can recruit the CREB/ATF-family transcription factor Atf1, then induce the expression of non-coding transcripts upstream of the *fbp1* gene, the so-called *mlonRNAs.* These metabolic stress-induced lncRNAs trigger the disruption of the chromatin structure around UAS2, which in turn allows for binding of the zinc-finger transcription factor Rst2 to UAS2. The chromatin around the TATA box of the *fbp1* gene is then modified and the stepwise chromatin remodeling through the *fbp1* promoter results in and the eventual full expression of *fbp1* in the absence of glucose. **(F)** The expression of the *FLO11* gene is indirectly controlled by two lncRNAs, *ICR1* and *PWR1*. The lncRNA *ICR1* (3.2 knt) is transcribed in the same direction as *FLO11* from the upstream intergenic region while *PWR1* (1.2 knt) is antisense to *ICR1*. Both Flo8 and Sfl1 compete for binding to the *FLO11* promoter, and also determining which of the two lncRNAs is expressed. histone deacetylase Hda1-mediated deacetylation condenses the chromatin at the Flo8 and Sfl1 binding sites. When Flo8 binding over Sfl1, the transcription of *PWR1* is activated then *PWR1* interferes with *ICR1* via transcriptional interference, leading to the expression of *FLO11*. Conversely, the binding of Sfl1 recruits histone deacetylase Hda1 and *PWR1* is not transcribed, enabling *ICR1* transcription to interfere with *FLO11*.

Interestingly, in contrast to *S. cerevisiae*, the meiosis mechanism in *S. pombe* is controlled by a lncRNA termed *meiRNA* with a different regulatory strategy. *meiRNA* is not involved in the induction of meiosis, but in meiotic progression and also chromosomal pairing (Ding et al., 2012). In meiotic cells, two isoforms of the *meiRNA* (*meiRNA-S* and *meiRNA-L*) are transcribed from the locus and physically interact with RNA-binding protein Mei2 to form a *meiRNA-*Mei2 complex (Figure 1D). In the nucleus, the Mei2-meiRNA complex blocks another RNA-binding protein, Mmi1, a crucial inhibitor of meiosis, thus allowing *sme2* to escape degradation and be translated in the cytoplasm, initiating meiosis. Moreover, a robustly *meiRNA-*dependent chromosome pairing at the *sme2* loci was also discovered (Figure 2). Interestingly, Mmi1 also inhibits Mei2 by forming a double-negative feedback loop (Shichino et al., 2014). In addition, Mmi1 interacts with a nuclear complex called Meiotic gene silencing complex MTREC/NURS MTREC (MTl1-REd1 Core) or NURS (NUclear RNA Silencing) that is involved in meiotic transcript elimination (Shichino et al., 2020).

## Metabolisms and Nutrition

### Phosphate Metabolisms

The fission yeast *S. pombe*, at least three phosphate acquisition genes, including a cell surface acid phosphatase *Pho1*, an inorganic phosphate transporter *Pho84*, and a glycerophosphate transporter *Tgp1*, have been identified to responsible for phosphate metabolisms (Carter-O’Connell et al., 2012). At least three lncRNAs have been reported to be transcribed under phosphate-rich conditions and repressed upon starvation, and they repress their sense-oriented target genes in *cis* through transcription interference (Ard et al., 2014; Chatterjee et al., 2016; Garg et al., 2018). The lncRNA *Pho1*-Repressing Transcript (*prt*) arises from the 1,147 nt upstream of the *pho1* mRNA transcription start site in response to the addition of phosphate. However, with the deprivation of phosphate, the expression of *prt* is abolished through an unknown mechanism, leading to the expression of *pho1* (Chatterjee et al., 2016). The lncRNA *nc-tgp1*, which originates at 1,823 nt upstream of the *tgp1* mRNA start site, is transcribed in the presence of phosphate. However, upon phosphate deprivation, the derepression of *nc-tgp1* leads to a decrease in the nucleosome occupancy, then result in the expression of *tgp1* (Ard et al., 2014). The expression of both *pho1* and *tgp1* mRNAs depends on the DNA-binding transcription factor Pho7. It can recognize a 12 nt sequence motif (5′- TCG(G/C)(A/T)xxTTxAA-3′) present in the *pho1* and *tgp1* promoters (Schwer et al., 2017; Garg et al., 2019). The transcription of *prt* and *nc-tgp1* may increase nucleosome density at the *pho1* and *tgp1* promoters, respectively, and displace Pho7 from the respective promoters (Figure 2A). Recently, it was reported that the dissociation of Pho7 from the *pho1* locus which results in the transcription of *prt* was governed not only by the lncRNA *prt* itself but also by RNAPII, depending on its phosphorylation status (Chatterjee et al., 2016). Changes in the phosphorylation status of RNAPII may lead to the early termination of *prt* transcription, resulting in the derepression of *pho1*. A similar mechanism has been described for *nc-tgp1* and its target gene *tgp1* (Ard et al., 2014; Sanchez et al., 2018). However, in addition to *prt*, another regulatory RNA, named *prt2*, controls the expression of *pho84* (Garg et al., 2018). Similar to the previously mentioned lncRNAs, *prt2* is transcribed upon phosphate starvation and regulate the repression of its neighbor gene *pho84* (Figure 2B). Moreover, the phosphorylation status of RNAPII impacts the expression of its target gene. Interestingly, *prt2* not only affects its adjacent gene *pho84*, but also influence the expression of the lncRNA *prt* and its regulated gene *pho1*. The inactivation of *prt2* leads to an upregulation of *pho84*, which consequently results in a downregulation of *prt*, and finally conduct to the transcription of *pho1* (Garg et al., 2018). Both *prt* and *nc-tgp1* are unstable owing to degradation by the nuclear exosome, and both carry a cluster of Mmi1-binding DSR (determinant of selective removal) motifs. The binding of Mmi1 in *prt* and *nc-tgp1* induces the assembly of heterochromatin at their target gene loci (Shah et al., 2014). However, the specific contributions of Mmi1 and the nuclear exosome involved in transcription interference remain to be elucidated. However, this mechanism is independent of the regulatory impacts on their adjacent genes (Ard et al., 2014; Chatterjee et al., 2016).

As in *S. pombe*, a short antisense transcript of *PHO84* also have been reported to affect the expression of *PHO84* in *S. cerevisiae*. The antisense transcript, which originates from *PHO84* locus, accumulates during aging, leading to the recruitment of the histone deacetylase complex Hda1/2/3 to the *PHO84* promoter, the deacetylation of H3K18, and the silencing of *PHO84* sense transcription (Camblong et al., 2007). These transcripts are degraded rapidly by the nuclear exosome subunit Rrp6. In Δ*Rrp6* cells during phosphate starvation, the induction of *PHO84* is delayed because of the lncRNA accumulation (Castelnuovo et al., 2013).

Interestingly, although the majority of antisense transcripts are generally involved in the anti-regulation of their sense strands, there are still rare examples of positive gene regulation through antisense transcripts. For example, the expression of *PHO5* is induced during phosphate starvation and shut-off in the presence of phosphate. A 2.4 kb antisense lncRNA activates *PHO5* transcription during phosphate starvation (Uhler et al., 2007). This lncRNA is initiated at the 3′ end of the *PHO5* gene, and it spans the *PHO5* open reading frame and its promoter region. This antisense transcript is repressed during phosphate starvation. The low expression level of the ncRNA allows histone eviction from the *PHO5* promoter and subsequent RNAPII recruitment, which enhances the initiation of *PHO5* transcription (Uhler et al., 2007).

### Galactose Utilization

The *GAL1-10* cluster of *S. cerevisiae* is tightly regulated by environmental sugar availability. In the presence of galactose and the absence of glucose, *GAL* genes are induced, while in the presence of glucose, they are repressed. This highly regulated nutrient-response system allows *S. cerevisiae* to thrive on a wide range of carbon sources. At least two lncRNAs have been characterized as being involved in galactose metabolism by regulating th*e GAL* cluster of genes. In 2008, Houseley et al. (2008) identified an antisense transcript *GAL10-*ncRNA that was initiated near the 3′ end of *GAL10* and terminated in the *GAL1*-coding region in the absence of galactose (a condition that represses *GAL10*). The transcription of this antisense transcript is associated with the transcriptional activator Reb1, which binds to chromatin near the site of lncRNA initiation (Figure 2C). The transcription of *GAL10*-ncRNA also needs the recruitment of Set2 methyltransferase and histone deacetylation activities in *cis*, leading to increased H3K4 di- and tri-methylation within the *GAL10*-coding region, increased H3K36 tri-methylation, and decreased H3 acetylation across the *GAL1-10* loci. This results in the silencing of all *GAL* loci (Houseley et al., 2008; Pinskaya et al., 2009). In addition, in the absence of galactose, although the Gal4 activator constitutively binds to *GAL* promoters, its activity is suppressed by Gal80 (Selleck and Majors, 1987). However, in the presence of galactose, *GAL* gene is activated with the release of Gal4. Both the *GAL3* and *GAL1* genes are activated by the Gal4 activator, forming two positive-feedback loops and leading to stable Gal4 release (Zenke et al., 1996; Acar et al., 2005; Venturelli et al., 2012). Furthermore, RNA degradation mechanisms also affect the expression of *GAL10*-ncRNA (Yoon et al., 2010; Figure 2C). In addition, the level of *GAL10-*lncRNA expression is elevated in the absence of the decapping enzyme Dcp2, and the degradation of *GAL10-*ncRNA is dependent on the cytoplasmic and nuclear 5’–3’ exonucleases Xrn1 and Rat1, respectively (Geisler et al., 2012). However, the deletion of *Xrn1* has a limited impact on *GAL1* expression. Another lncRNA termed *GAL4-*lncRNA has also been reported to regulate the expression of the transcriptional activator *GAL4*, although its mechanism has not been clarified in detail (Geisler et al., 2012).

### Serine Utilization

In media containing serine, the lncRNA *SRG1* is transcribed under serine-rich conditions, and it regulates the expression of its adjacent *SER3* gene that encodes a phosphoglycerate dehydrogenase involved in serine biosynthesis (Martens et al., 2004). This is the first example of non-coding transcriptional interference in yeast. When serine is available to the cells, *SRG1* transcription is turned on through a serine-dependent activator, Cha4, that recognizes an upstream activating sequence (UAS) in the *SRG1* promoter (Martens et al., 2005). Moreover, a coactivator complex, namely SAGA, and the ATPase subunit of the Swi/Snf chromatin-remodeling complex is recruited to initiate *SRG1* transcription, leading to the derepression of *SER*3 by increasing nucleosome occupancy at the SER3 promoter (Martens et al., 2005; Figure 2D). Recently, it was revealed that the transcription of *SRG1* also requires the involvement of the transcription elongation complex FACT (Facilitates Chromatin *Transcription*), the Paf1 complex, chromatin reassembly factors (Spt6, Spn1, and Spt2), and specific amino acids in histones H3 and H4 (Martens et al., 2005; Pruneski et al., 2011; Thebault et al., 2011; Hainer et al., 2012; Hainer and Martens, 2016). In the absence of FACT and Spt6/Spn1, the density of nucleoso—mes over the *SER3* promoter region was decreased but have no impact on *SRG1* transcription (Hainer et al., 2011). In the absence of serine, although the Cha4 is constitutively bound to the lncRNA promoter, its transcription is repressed and the *SER3* is depleted of nucleosomes, allowing both TBP and RNAPII and/or other unknown activator to bind and activate *SER3* transcription (Figure 2D; Hainer et al., 2011).

### Glucose Starvation

In the fission yeast *S. pombe*, glucose starvation induces the expression of the *fbp1* gene that encodes a fructose-1,6-bis phosphatase (Hirota et al., 2008). There are two cis-acting regulatory elements in the upstream region of *fbp1*: UAS1 and UAS2. During glucose starvation, UAS1 recruits the transcription factor Atf1 and then induces the expression of non-coding transcripts upstream of the *fbp1* gene, the so-called *mlonRNA*s (Figure 2E). These metabolic stress-induced lncRNAs allows the zinc-finger transcription factor Rst2 binds to UAS2 by disrupting the chromatin structure around UAS2 (Hirota et al., 2008; Figure 2E). The chromatin remodeling around the TATA box of the *fbp1* promoter results in the full expression of *fbp1* in the absence of glucose (Hirota et al., 2008). In addition, both the *mlonRNAs* and their antisense transcripts are degraded by the nuclear exosome/Rrp6 complex (Galipon et al., 2013; Miki et al., 2016).

### Nitrogen Starvation

In *S. cerevisiae*, *ASP3* encodes an enzyme Asparaginase II that can hydrolyze both D- and L-asparagine to aspartate and ammonium cations (Dunlop et al., 1978). *ASP3* is activated in a nitrogen-limited environment (Dunlop et al., 1980; Kim et al., 1988). The lncRNA *ncASP3*, an intragenic sense-oriented transcript within the *ASP3* coding region, is expressed when nitrogen is either available or depleted. The continuous expression of *ncASP3* results in a high level of trimethylation of histone H3 at lysine 4 (H3K4me3) at the *ASP3* promoter and makes this region more accessible for RNAPII’s transcription (Huang et al., 2010).

## Cell-Cell Adhesion

In *S. cerevisiae*, the *FLO11* gene encodes a cell wall glycoprotein that controls cell-cell adhesion, and only cells expressing *FLO11* can undergo pseudohyphal growth (Halme et al., 2004). Research showed that the expression of the *FLO11* gene is indirectly controlled by two lncRNAs, *ICR1* and *PWR1*, forming a complicated regulatory model of *cis*-acting lncRNAs in fungi (Figure 2F). The lncRNA *ICR1* (3.2 kb) is transcribed from the upstream intergenic region of *FLO11* in the same direction while *PWR1* (1.2 kb) is antisense to *ICR1*. The expression of *FLO11* is repressed when the transcription factor Sfl1 binds to the *FLO11* promoter, while another transcription factor, Flo8, activates it. Both Flo8 and Sfl1 compete for *FLO11* promoter binding, and which of the two lncRNAs is expressed is determined through histone deacetylase Hda1 regulation. Hda1-mediated deacetylation condenses the chromatin at the Flo8- and Sfl1-binding sites. When Flo8 binds Sfl1, the transcription of *PWR1* is activated. Then, *PWR1* interferes with *ICR1* through transcriptional interference, leading to the expression of *FLO11*. Conversely, the expression of *ICR1* is activated with the binding of Sfl1 and the recruitment of histone deacetylase Hda1, then prevent the transcription of *FLO11* (Figure 2F; Bumgarner et al., 2009).

## Circadian Rhythm Maintenance

In *N. crassa*, the circadian clock core regulatory gene *frq* generates sustained rhythmicity (Gardner and Feldman, 1980). The long non-coding *qrf*, the antisense transcript of *frq*, is required for the rhythmic conidiation of the fungus. The transcription of *qrf* affects the clock’s response to light through chromatin modifications at the *frq* promoter (Kramer et al., 2003; Belden et al., 2011). The transcription of *qrf* represses *frq* expression by mediating chromatin modifications and the prior termination of transcription, and it regulates clock resetting. However, *frq* transcription also inhibits *qrf* expression and drives the antiphasic expression of *qrf*. Thus, the transcription of *frq* and *qrf* forms mutual inhibition of a double-negative feedback loop that is interconnected with the core feedback loop (Xue et al., 2014).

## Stress Response

In *S. cerevisiae*, the modulation of cell-cycle control is controlled by the stress-activated protein kinase p38/Hog1. Upon osomostress, a large set of lncRNAs, including the antisense lncRNA of *CDC28*, is induced by Hog1 to regulate the expression of the master cell cycle regulator CDK1/Cdc28 (Nadal-Ribelles et al., 2014). Through increasing the Cdc28 level, the *CDC28* lncRNA promotes the stressed cells efficient enter into the cell cycle. The antisense lncRNA is induced when Hog1 is associated with the 3′ region of *CDC28*. Then forms a gene looping between the 5′- and 3′- UTRs of *CDC28*, which causes the relocation of Hog1 to the 5′ region. The relocated Hog1 then promotes chromatin remodeling by recruiting the RNA-induced silencing remodeling complex and inducing *CDC28* expression (Nadal-Ribelles et al., 2014).

In addition, another lncRNA*SPNCRNA.1164* has also been reported to regulate the expression of a stress-responsive transcription factor, Atf1, under oxidative stress conditions, although its mode of action remains unknown (Leong et al., 2014). Glucose starvation cannot induce the expression of *SPNCRNA.1164*, suggesting that it may be respond only to specific stress stimuli (Leong et al., 2014).

## Telomere Maintenance

In *S. cerevisiae*, two sorts of telomere-associated lncRNAs have been identified: *TERRA* and the telomerase RNA *TCL1*. *TERRA* is an evolutionary conserved lncRNA that has been found in many eukaryotic cells, such as those of humans (*Homo sapiens*), budding yeast (*S. cerevisiae*), fission yeast (*S. pombe*), mice (*Mus musculus*), zebrafish (*Danio rerio*), and plants (*Arabidopsis thaliana*), indicating its important functions in regulating telomerase (Azzalin et al., 2007; Luke et al., 2008; Azzalin and Lingner, 2015). During the telomeric synthesis process, *TERRA* forms a scaffold that connects both the telomeric DNAs and chromatin-modifying enzymes to maintain telomerase activity (Luke et al., 2008). In addition, *TERR*A interacts with other associated telomeric proteins to regulate the integrity of the telomere. In yeast, *TERRA* is regulated by the 5′–3′ exonuclease, Rat1p and stabilized by Pap1p. In the mutant rat1-1 cells, *TERRA* is accumulated and harbored in short telomeres because of defects in telomerase-mediated telomere elongation (Cusanelli and Chartrand, 2015).

Another telomere-associated lncRNA described in *S. cerevisiae* is *TLC1*. This lncRNA is transcribed on chromosome II and has a secondary structure composed of three long arms (Singer and Gottschling, 1994). *TLC1* form the telomerase complex by physically interacting with the proteins Est1, Est2, and Est3. This complex involves in the telomeric DNA repeats synthesis and prevent telomere-shortening cell division (Gallardo et al., 2008). In *S. pombe*, a homolog of *TLC1*, named *TER1*, has also been discovered. In comparison with *TLC1*, *TER1* is much larger and contains more invariant repeats (Leonardi et al., 2008).

## Pathogenicity

Although there only a few fungal lncRNAs related to pathogenicity has been reported, we still believe that lncRNAs derived from pathogenic fungi may play important roles during fungi-host interaction. These lncRNAs which is involved in the pathogenicity may enhance the virulence of fungi or they can repress host immune response during infection. In the biotrophic basidiomycete fungus *Ustilago maydis*, RT-PCR showed that *ncrna1*, an antisense transcript complementary to the 3′-UTR of *um02150*, is expressed during corn infection. The infected ability was reduced with the absence of *ncrna1*, suggesting that *ncrna1* may be involved in pathogenesis though the mechanism has yet to be determined (Morrison and Saville, 2012). Also in *U. maydis*, the expression of the natural antisense transcript *as-um02151* results in a twofold increase in complementary mRNA levels. The alteration of its expression decreases pathogenesis too (Donaldson and Saville, 2013). However, both detailed regulatory mechanisms of these two putative lncRNAs which may relate to pathogenesis have not been identified yet. Recently, Tang et al. elucidated the lncRNA profiles during infection and development of phytopathogen *Ustilaginoidea virens*. RNA-seq analyses revealed more than 1700 lncRNAs in *U. virens* (Tang et al., 2021). However, functions of these lncRNAs still undetermined yet.

## Other Functions

In 2008, Berretta et al. found that several antisense ncRNAs were transcribed within *Ty1* elements in *S. cerevisiae*, and function in *trans* (Berretta et al., 2008). The expression of those antisense ncRNA may conduct to the suppression of *Ty1* elements in *trans* and also lead to the repression of the *Ty1* promoter and 5’ long terminal repeat, which suggests that antisense ncRNAs regulate *Ty1* in *trans.* These ncRNAs hold at very low levels because of the RNA surveillance mechanisms. Additionally, Set1 is required for the silencing of *Ty1* elements by antisense *Ty1* transcripts (Berretta et al., 2008). In *S. cerevisiae*, the protein-coding sense gene *MDF1* significantly suppresses the mating efficiency in a rich medium by binding *MATα2*, thereby promoting vegetative growth. The antisense gene *ADF1* acts as a transcriptional suppressor that regulates the expression of the sense gene *MDF1* (Li et al., 2010).

In addition, besides the regulatory lncRNA which was functionally identified in yeast, several lncRNAs have also been characterized in filamentous fungi. For instance, the deletion of the poorly transcribed lncRNA *carP* (*Fo-carP* and *Ff-carP* in the fungi *Fusarium oxysporum* and *F. fujikuroi*, respectively) leads to strong reductions of the carotenoid biosynthesis genes at mRNA levels. However, the content of *carS* mRNA is maintained at a higher level, indicating that *carP* is involved in activating the expression of carotenoid genes in *Fusarium* with an unknown molecular mechanism (Parra-Rivero et al., 2020). Additionally, in the fungal pathogen *Cryptococcus neoformans*, the lncRNA *RZE1* controls the yeast-to-hyphal transition by regulating the key morphogenesis regulator Znf2 (Chacko et al., 2015). Moreover, a lncRNA termed *HAX1* has been identified as promoting cellulase expression in *Trichoderma reesei* (Till et al., 2018). In the maize pathogen *Cochliobolus heterostrophus*, the transcription factor Cmr1, which regulates melanin biosynthesis, is transcribed in both sense and antisense directions. The transition of the melanin gene cluster is regulated by both the antisense transcript of Cmr1 and the MAPK pathway, resulting in the euchromatin and heterochromation (Eliahu et al., 2007).

## Conclusion and Perspectives

With the availability of high-throughput sequencing technology, the identification of lncRNAs is becoming easier. Thus, scientists are facing another major challenge: the functional characterization of the regulatory mechanisms of ncRNAs. However, owing to the non-conservative, development- and condition-specific nature of lncRNAs, it is still difficult to identify their functions at the molecular level. For example, although the sporulation processes of the two yeasts *S. cerevisiae* and *S. pombe* are both controlled by lncRNA-based chromatin silencing, their regulatory mechanisms are totally divergent. This suggested that there is great diversity in regulatory mechanisms in fungi kingdom. Therefore, more extensive studies are still needed to clarify the molecular mechanisms of expression, regulation, and functions of lncRNAs. Moreover, functional identifications in other fungi, especially pathogenic fungi, will result in a comprehensive understanding of pathogenicity mechanisms in fungi. However, pathogenicity-related lncRNAs in fungi is still less well documented. Advanced sequencing data will unveil profiles of lncRNAs and provide new insights and promising lncRNA candidates in this area.

Recently, a new type of lncRNA, circRNA, has been identified as playing important roles in animals and plants (Kristensen et al., 2019), but little attention has been focused on fungal circRNAs. Although Yuan et al. (2018) identified more than 8,000 circRNAs from the model plant-pathogenic fungus *M. oryzae*, and these circRNAs may play important roles in the regulation of growth and development. Thus, there is enormous potential for increasing our understanding of the relatively new field of circRNAs in fungi.

We believe that ongoing fungal lncRNA-based studies will provide important insights into how lncRNAs regulate important biological processes in fungi. Research in simple eukaryotes may provide important clues for understanding how this novel discovered regulatory lncRNAs function in eukaryotic cells.

## Author Contributions

JL conceived the manuscript. JL, XL, ZY, ZH, and K-QZ discussed and developed its conceptual framework. JL wrote the manuscript. All authors contributed to the article and approved the submitted version.

## Conflict of Interest

The authors declare that the research was conducted in the absence of any commercial or financial relationships that could be construed as a potential conflict of interest.
